# Zonulin Family Peptide Levels in Ascites and Serum in Patients with Liver Cirrhosis: A Preliminary Study

**DOI:** 10.1155/2019/2804091

**Published:** 2019-11-05

**Authors:** Marta Pietrukaniec, Maciej Migacz, Agnieszka Żak-Gołąb, Magdalena Olszanecka-Glinianowicz, Jerzy Chudek, Jan Duława, Michał Holecki

**Affiliations:** ^1^Department of Internal, Autoimmune and Metabolic Diseases, School of Medicine in Katowice, Medical University of Silesia, Katowice, Poland; ^2^Department of Pathophysiology, School of Medicine in Katowice, Medical University of Silesia, Katowice, Poland; ^3^Department of Internal Diseases and Oncological Chemotherapy, School of Medicine in Katowice, Medical University of Silesia, Katowice, Poland; ^4^Department of Internal and Autoimmune Diseases, School of Health Science, Medical University of Silesia, Katowice, Poland

## Abstract

**Introduction:**

Zonulin is a protein that reversibly modulates the permeability of tight junction of the small intestine wall. As the serum concentration of “zonulin family peptides” (ZFPs) is considered to be a sensitive and useful marker of intestinal wall permeability, its serum level may affect the volume of ascites fluid and change in gut microbiota. The aim of the study was to assess the association between concentrations of ZFPs in serum and ascites in relation to the severity of liver cirrhosis.

**Methods:**

The preliminary study included 24 adult patients diagnosed with alcoholic or viral liver cirrhosis. 18 healthy adult subjects were enrolled as the control group. In patients and controls, there were measured serum and ascites (only in patients) ZFPs, serum bilirubin, creatinine, alanine aminotransferase, total protein, and C-reactive protein (CRP).

**Results:**

Cirrhotic patients had lower serum hemoglobin (11.6 vs. 14.3 mg/dL; *p* < 0.001), platelet count (178 vs. 305 × 10^3^/mm^3^; *p* < 0.01), total protein and albumin (58.6 vs. 74.3 g/dL; *p* < 0.001, 26.6 vs. 42.3 g/dL; *p* < 0.001, respectively), and serum ZFPs (30.5 vs. 62.0 ng/mL; *p* < 0.001) in comparison to controls. In patients with cirrhosis serum bilirubin, C-reactive protein level and INR were higher than in controls (3.07 vs. 0.96 mg/dL; 36.9 vs. 5 mg/L; 1.53 vs. 0.95; *p* < 0.001, respectively). Patients with low ZFP levels were characterized with lower ascites ZFP levels (0.25 vs. 16.4 ng/mL; *p* < 0.001) and ascites/serum index (0.011 vs. 0.462; *p* < 0.001). There were negative correlations between ascites ZFPs and platelet count (*R* = −0.497; *p* < 0.01) and positive correlation with INR (*R* = 0.640; *p* < 0.001). ZFP index positively correlated with platelet count (*R* = 0.726; *p* < 0.001) and negatively with INR (*R* = −0.392; *p* = 0.06).

**Conclusions:**

Decrease serum ZFP levels seem to reflect their decreased liver synthesis but not increased gut permeability in patients with liver cirrhosis. The physiologically low level of ZFPs in transudate is increased in exudate.

## 1. Introduction

Zonulin, primarily discovered in 2000 by Fasano [1], is a 47 kDa protein that reversibly modulates (increases) the permeability of tight junction of the small intestine wall and participates in the development of intestinal innate immunity. This protein, which synthesizes in intestinal and liver cells, activates the target receptor in the intestinal wall in a manner similar to the ZOT toxin (zonula occludens toxin) produced by the cholera cutter. Increased concentration of serum zonulin is a sensitive indicator of the increased permeability of the intestinal wall and has been observed to be upregulated in several autoimmune diseases, including with celiac disease, type 1 diabetes, nonalcoholic fatty liver disease, rheumatoid arthritis, diabetes, and multiple sclerosis [2–4].

In the intestinal mucosa, zonulin is considered to inhibit the entry of bacteria, antigens, toxins, and other pathogens while absorption of necessary nutrients is not impacted. In some clinical conditions the harmful agents may leak into the circulation due to increased intestinal permeability. The linkage between zonulin level and gut microbiota has already been described by Zak-Gołąb et al., who observed interrelation between selected inflammatory markers and serum zonulin [5]. In addition, impaired integrity of the intestinal wall may be an important risk factor for spontaneous peritonitis in patients with liver cirrhosis.

The zonulin sequence established by Wang et al. and Di Pierro et al. [6, 7] was used to develop the polyclonal antibody applied in the Immundiagnostik AG ELISA. Scheffler et al. [8] revealed that the ELISA does not detect zonulin (the recombinant pre-Hp-2) but properdin—an activator of the alternative complement pathway. According to Fasano comment, properdin is functionally and structurally belonging to the “zonulin family”—permeability-increasing mediators [9].

As the serum concentration of “zonulin family peptides” (ZFPs) is considered to be a sensitive and useful marker of intestinal wall permeability, its serum level may affect the volume of ascites fluid and change in gut microbiota. To our best knowledge, in the previously published data on ZFPs, the association of these peptide levels in ascites fluid with the liver cirrhosis has not been investigated. Thus, the aim of the study was to assess the association between concentrations of ZFPs in serum and ascites in relation to the severity of liver cirrhosis.

## 2. Materials and Methods

The preliminary study included 24 adult patients (12 males and 12 females; age range 34-93) diagnosed with alcoholic (regardless of keeping the abstinence) or viral liver (chronic hepatitis B without history of antiviral therapy) cirrhosis who has been hospitalized at the department of internal medicine due to progressive and painful abdominal distension due to massive ascites that required paracentesis. We did not collect detailed information why the patients with hepatitis B were not receiving antiviral therapy (decompensated liver cirrhosis is considered as the exclusion criterion for antiviral therapy in the drug program covered by the Polish National Health Fund).

We did exclude both active or subclinical infections, hepatocellular carcinoma, and bleeding. There was history of upper gastrointestinal bleeding in one-third of the study group. In addition, more than half of the patients (*N* = 14) had been previously provided with paracentesis.

18 healthy adult subjects (6 males and 12 females, age range 30-70 years) were enrolled as the control group. Patients' characteristics are presented in Table 1.

The research protocol was approved by the local Ethics Committee of the Medical University of Silesia in Katowice. All clinical investigation was conducted according to the principles expressed in the Declaration of Helsinki. Signed informed consent was obtained from all subjects who participated in the study.

Patients and controls were subjected to complete history taking along with thorough clinical examination and laboratory investigations. Blood samples were taken from each subject, after an overnight fast, for estimation of serum and ascites (only in patients) ZFPs, serum bilirubin, creatinine, alanine aminotransferase, total protein, and C-reactive protein (CRP). The serum and ascites fluid samples were stored in -70°C until the time of the assay.

### 2.1. Laboratory Analyses

Serum “zonulin family peptide” (ZFP) levels were measured by an ELISA technique used for the quantitative determination of human ZFPs in serum, plasma, and tissue homogenates. The kit was provided by Immundiagnostik AG (Bensheim, Germany).

Serum bilirubin, creatinine, alanine aminotransferase, total protein, and C-reactive protein were assessed by an automated system in a single-certified laboratory.

### 2.2. Data Analysis

The Model for End-Stage Liver Disease (MELD) score was calculated according to standard formula, as follows: MELD = 3.78 × ln[serum bilirubin (mg/dL)] + 11.2 × ln[INR] + 9.57 × ln[serum creatinine (mg/dL)] + 6.43 [10].

The classification of ascites fluid (transudate vs. exudate) was based on protein content < 1 g/dL or >1 g/dL, according to Polish recommendation [11].

### 2.3. Statistics

The analysis was performed using the MedCalc 14.8.1 licensed software (MedCalc Software, Ostend, Belgium). Quantity variables were presented as means with 95% confidence interval or medians with interquartile range (1-3Q), due to nonparametric distribution on numerous parameters. Quality variables were presented as an absolute value and the proportion.

The intersubgroup differences were assessed with the Student *t*-test and the Mann-Whitney *U* tests, when appropriate. The chi-square test was used for quality variables. Correlation coefficients were calculated according to Spearman. The statistical significance was established at *p* values below 0.05.

## 3. Results

The clinical characteristics of the patients and controls are summarized in Table 1. Significant differences were observed in age, hemoglobin, serum protein, albumin, CRP, bilirubin, “zonulin family peptide” levels, platelet count, and INR. Cirrhotic patients had lower serum hemoglobin (11.6 vs. 14.3 mg/dL; *p* < 0.001), platelet count (178 vs. 305 × 10^3^/*μ*L; *p* < 0.01), total protein and albumin (58.6 vs. 74.3 g/dL; *p* < 0.001, 26.6 vs. 42.3 g/dL; *p* < 0.001, respectively), and serum ZFPs (30.5 vs. 62.0 ng/mL; *p* < 0.001) in comparison to controls.

In patients with cirrhosis serum bilirubin, C-reactive protein level and INR were higher than in controls (3.07 vs. 0.96 mg/dL; 36.9 vs. 5 mg/L; 1.53 vs. 0.95; *p* < 0.001, respectively).

**Figure 1:**
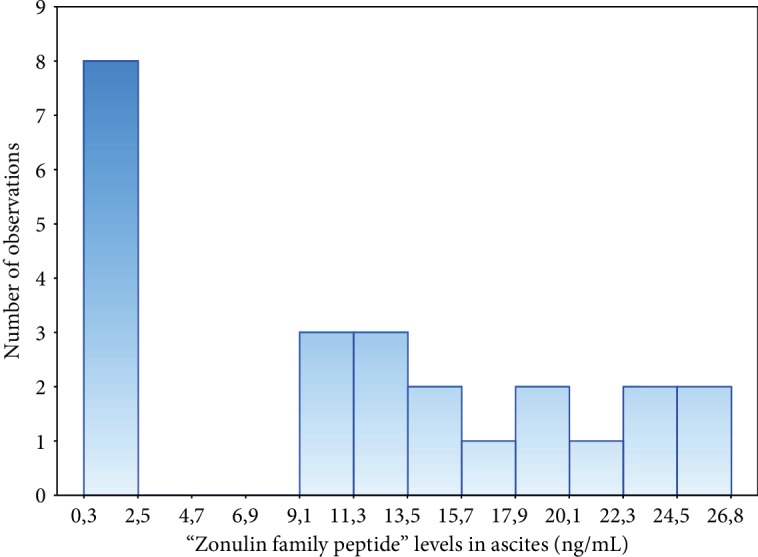
The levels of “zonulin family peptides” (ZFPs) in ascites in patients with liver cirrhosis.

The levels of “zonulin family peptides” in ascites were only measured in the study group. There was a clear separation into two subgroups: with levels at least 50 times lower than in the serum and with a few times lower levels (Figure 1).

After being divided according to the zonulin serum level (low vs. high)—Table 2, patients with low ZFP levels were characterized with lower ascites ZFP levels (0.25 vs. 16.4 ng/mL; *p* < 0.001) and ascites/serum index (0.011 vs. 0.462; *p* < 0.001). There were no differences in other biochemical parameters, age, sex, etiology of the ascites, the Child-Pugh score, and the MELD score.

There were also neither differences when divided into subgroups according to the etiology of the ascites (transudate vs. exudate) nor in the MELD score—data presented in Table 3.

There were negative correlations between ascites ZFPs and platelet count (*R* = −0.497; *p* < 0.01) and positive correlation with INR (*R* = 0.640; *p* < 0.001). ZFP index positively correlated with platelet count (*R* = 0.726; *p* < 0.001) and negatively with INR (*R* = −0.392; *p* = 0.06). There were no correlations between both serum and ascites ZFPs, as well as ZFP index and MELD score.

## 4. Discussion

As “zonulin family peptides” (ZFPs), that regulates intestinal permeability, are being produced both by intestinal and liver cells, we focused on its serum level in patients with liver cirrhosis, as we supposed that it may reflect the permeability of the gut. Additionally, we measured ZFP levels in fluid evacuated after paracentesis as a potential surrogate of leaky gut and permeability of blood vessels in the splanchnic system. To our best knowledge, this is probably the first study that evaluated zonulin family peptides' levels both in serum and ascites fluid.

Mild liver injury is associated with higher serum levels of ZFPs, as described in patients with fatty liver disease [12]. While in both severe liver injury (hepatitis) and cirrhotic patients, serum levels of ZFPs are significantly decreased as compared to controls. Akao and colleagues have described decreased ZFP levels both in patients with viral B and C hepatitis [3]. However, the authors did not measure levels of ZFPs in ascitic fluid. We performed such measurements in two ascitic subgroups, one with ascitic fluid ZFPs at least fifty times lower compared to serum levels and the second with several-fold difference between ascitic and serum ZFPs. It should be noted that the subgroups did not differ in other biochemical parameters, age, sex, etiology of the ascites, and the Child-Pugh score. The meaning of our finding is unclear. If the lower serum level of zonulin in cirrhotic patients could be explained by the degree of hepatocyte damage, it is hard to believe that higher volume of fluid would be associated with a lower level of ZFPs in the fluid due to intestinal cell disruption. The more, so since the ascitic fluid volume is generally dependent on the degree of portal hypertension and serum albumin. It is not clear whether it is also associated with decreased liver production or additionally with disrupted intestinal barrier. The former is much more likely, as we did not find any correlation except those associated with platelet count and INR, of which indirectly evidence liver damage. On the other hand, Handy et al. observed a significant progressive increase in the serum ZFP level in patients with more pronounced liver damage (from control to simple steatosis to NASH, respectively) [13]. These results are in contrast to our hypothesis. However, it is also possible that the ZFP level may be differently related to a different clinical condition or, according to Akao et al., its functional activity as well as its production by the liver should be clearly dissected. Thus, there may be a discrepancy between the level of ZFPs and its functional implications.

It is worth noting that Raparelli and colleagues observed higher levels of serum zonulin/ZFPs in cirrhotic patients versus controls, using, recently developed by Elabscience (USA), the ELISA kit [14]. Therefore, we cannot be sure that both methods used by us and Raparelli et al. are able to measure the same epitopes/molecules. It seems that rather not, as the detected levels markedly differs. Of note, the cited study did not show any differences in zonulin/ZFP levels across Child-Pugh classes.

The main limitation of our study is a relatively small group of patients and the lack of pressure measurement in the hepatic portal vein; however, this is the first one that documented preliminary data concerning ZFP levels in ascites in patients with liver cirrhosis independently of its serum levels.

## 5. Conclusions

(1) Decrease serum “zonulin family peptide” levels seem to reflect their decreased liver synthesis but not increased gut permeability in patients with liver cirrhosis. (2) The physiologically low level of “zonulin family peptides” in transudate is increased in exudate.

## Figures and Tables

**Table 1:** Characteristics of the study group and the controls. Data is presented as mean value with 95% confidence interval or median with quartiles (1-3Q)^∗^.

Parameters	Study group *N* = 24	Controls *N* = 18	*p* value
Age (years)	68 (61-75)	42 (35-49)	<0.001
Sex (men/women)	11/13	4/14	0.12
Hb (g/dL)	11.6 (10.4-12.7)	14.3 (13.9-14.7)	<0.001
PLT (10^3^/*μ*L)^∗^	178 (114-282)	305 (253-370)	<0.01
WBC (10^3^/*μ*L)	10.2 (8.0-12.3)	7.2 (5.9-8.5)	0.03
ALT (U/L)	24.3 (17.2-31.5)	27.9 (25.2-30.6)	0.39
INR^∗^	1.53 (1.15-1.99)	0.95 (0.80-1.00)	<0.001
Serum protein (g/dL)	58.6 (56.1-61.2)	74.3 (71.7-76.9)	<0.001
Serum albumin (g/dL)	26.6 (24.1-29.2)	42.3 (39.7-44.9)	<0.001
C-reactive protein (mg/L)^∗^	36.9 (20.8-59.0)	<5	<0.001
Serum creatinine (mg/dL)	1.56 (0.92-2.21)	0.86 (0.78-0.94)	0.06
Serum bilirubin (mg/dL)	3.07 (1.94-4.20)	0.96 (0.89-1.02)	< 0.001
Serum ZFPs (ng/mL)^∗^	30.5 (23.6-35.6)	62.0 (47.6-67.4)	<0.001
Ascites ZFPs (ng/mL)^∗^	12.1 (0.25-18.8)	—	—

ZFPs: zonulin family peptides.

**Table 2:** Comparison of patients with low and high “zonulin family peptides” in ascites. Data is presented as mean value with 95% confidence interval or median with quartiles (1-3Q)^∗^.

Parameters	Low ZFP levels *N* = 8	High ZFP levels *N* = 16	*p* value
Age (years)^∗^	62 (52-84)	70 (59-79)	0.70
Sex (men/women)	3/5	8/8	0.57
Hb (g/dL)^∗^	9.8 (7.8-13.2)	13.1 (11.4-13.6)	0.21
PLT (10^3^/*μ*L)^∗^	130 (107-178)	218 (156-319)	0.06
WBC (10^3^/*μ*L)^∗^	10.5 (5.1-12.6)	9.0 (5.9-12.5)	0.83
ALT (U/L)^∗^	21.5 (14.0-43.5)	19.5 (10.0-31.5)	0.38
Serum protein (g/dL)^∗^	58.8 (58.0-63.5)	58.0 (53.1-62.7)	0.61
Serum albumin (g/dL)^∗^	26.8 (26.0-31.5)	26.0 (21.1-30.9)	0.61
C-reactive protein (mg/L)^∗^	22.4 (21.2-50.9)	41.0 (26.5-118.7)	0.11
Serum creatinine (mg/dL)^∗^	1.25 (0.55-1.45)	1.35 (0.84-1.70)	0.38
Serum bilirubin (mg/dL)^∗^	2.34 (1.67-5.12)	2.39 (0.89-4.03)	0.42
Serum ZFPs (ng/mL)^∗^	23.3 (17.9-27.0)	33.5 (29.7-43.6)	<0.001
Ascites ZFPs (ng/mL)^∗^	0.25 (0.25-0.27)	16.4 (12.1-21.6)	<0.001
Ascites/serum ZFPs^∗^	0.011 (0.010-0.014)	0.462 (0.387-0.569)	<0.001
Transudate/exudate (*n*)	6/2	7/9	0.16
The causes of liver cirrhosis (*n*)			
Inflammatory	2	0	
Alcoholic	5	6	0.02
Mix	1	1	
Unknown	0	9	
Child-Pugh score (*n*)			
Class A	1	0	
Class B	3	9	0.30
Class C	4	7	
MELD score (pts)	19.5 (10.0-29.1)	20.5 (14.7-26.3)	0.85
MELD score > 20 pts (*n*)	3 (37%)	8 (50%)	0.56

**Table 3:** Comparison of patients with transudate and exudate in the course of liver cirrhosis. Data is presented as mean value with 95% confidence interval or median with quartiles (1-3Q)^∗^.

Parameters	Transudate *N* = 13	Exudate *N* = 11	*p* value
Age (years)^∗^	72 (59-82)	67 (55-81)	0.57
Sex (men/women)	7/6	4/7	0.40
Hb (g/dL)^∗^	12.7 (10.5-13.5)	13.1 (9.3-13.7)	0.91
PLT (10^3^/*μ*L)^∗^	159 (109-196)	240 (119-406)	0.23
WBC (10^3^/*μ*L)^∗^	8.7 (5.0-11.2)	10.9 (6.6-17.1)	0.28
ALT (U/L)^∗^	19.0 (10.0-24.0)	23.0 (11.0-35.0)	0.73
Serum protein (g/dL)^∗^	58.0 (55.0-62.3)	58.0 (54.2-66.0)	1.0
Serum albumin (g/dL)^∗^	26.0 (23.0-30.3)	26.0 (22.2-34.0)	1.0
C-reactive protein (mg/L)^∗^	28.3 (16.0-37.6)	54.1 (36.2-172.5)	0.02
Serum creatinine (mg/dL)^∗^	1.30 (0.70-1.50)	1.40 (0.80-1.60)	0.65
Serum bilirubin (mg/dL)^∗^	2.04 (1.30-4.54)	3.00 (0.59-4.39)	0.78
Serum ZFPs (ng/mL)^∗^	30.2 (22.6-33.3)	31.6 (23.9-36.6)	0.39
Ascites ZFPs (ng/mL)^∗^	9.9 (0.25-15.0)	14.0 (10.5-22.4)	0.05
Ascites/serum ZFP^∗^	0.223 (0.013-0.450)	0.460 (0.368-0.666)	0.07
The causes of liver cirrhosis (*n*)			
Inflammatory	1	1	
Alcoholic	6	5	0.56
Mix	2	0	
Unknown	4	5	
Child-Pugh score (*n*)			
Class A	1	0	
Class B	6	6	0.63
Class C	6	5	
MELD score (pts)	21.2 (15.0-27.4)	19.0 (11.0-26.9)	0.63
MELD score > 20 pts (*n*)	6 (46.2%)	5 (45.5%)	0.56

## Data Availability

The data used to support the findings of this study are included within the article.
